# The efficacy of statins for improving cognitive impairments in pediatric patients with neurofibromatosis type 1 (NF-1): a meta-analysis

**DOI:** 10.3389/fped.2023.1274972

**Published:** 2023-10-09

**Authors:** Lutong Gan, Weiwen Zhu, Pengqing Fu

**Affiliations:** ^1^ Department of Neurology, The Second Hospital of Guangzhou Medical School of China, Guangzhou, China; ^2^ Department of Cardiology, The Second Hospital of Guangzhou Medical School of China, Guangzhou, China

**Keywords:** statins, neurofibromatosis type 1, cognitive function, children, systematic review

## Abstract

**Background:**

Given the considerable discrepancies in the evidence concerning the efficacy of statins in ameliorating cognitive impairments in pediatric patients with Neurofibromatosis Type 1 (NF-1), this study conducts a systematic review and meta-analysis to consolidate existing evidence to evaluate the efficacy of statins on cognitive impairments in children with NF-1.

**Methods:**

This study adhered to the PRISMA statement, and the research protocol was pre-registered on PROSPERO (#CRD: 42022369072). Comprehensive searches of databases including PubMed, Embase, and the Cochrane Library were performed up to March 31, 2023 to identify randomized controlled trials (RCTs) investigating the effects of statins on cognitive impairments in children with NF-1. Statistical analyses were conducted using Review Manager 5.4.1. A fixed- or random-effects model was employed according to the *I*^2^ statistic. As all data were continuous, MD [95% CI] was used as the pooled estimate.

**Results:**

The final analysis included five RCTs with a total of 364 patients. The meta-analysis indicated that aside from a statistically significant improvement in internalizing problems (MD [95%CI] = 3.61[0.11, 7.10], *p* = 0.04), Object assembly Test (MD [95%CI] = 0.53[0.12, 0.93], *p* = 0.01), Cancellation Test (MD [95%CI] = 3.61[0.11, 7.10], *p* < 0.0001), statins did not exhibit significant efficacy in improving other cognitive aspects in children with NF-1 (*p *> 0.05). An additional descriptive analysis on indices that cannot be meta-analyzed revealed considerable inconsistency in the therapeutic effect of statins across different studies.

**Conclusion:**

Current evidence suggests that statins may not be effective for cognitive performance in children with NF-1.

## 1. Introduction

Neurofibromatosis Type 1 (NF1) is a common multifaceted neurogenetic disorder as a result of germline mutations in one of the two alleles of the *NF1* tumor suppressor gene located on chromosome 17q11.2 (1–4). NF1 gene pathogenic variant deactivate the negative regulatory protein family (GTPase-activating proteins) on neurofibromin, a structural domain of 300 residues that functionally mirrors the RAS oncogene. This causes an overstimulation of Ras (p21Ras pathway), driving increased cell growth and survival, which ultimately induces NF1 (5–10). The prevalence of NF1 is approximately 1:2,000 to 1:3,000, with a characteristic autosomal dominant inheritance pattern (11, 12). Dermatological signs are integral to the NF1 diagnosis, inclusive of café-au-lait macules (CALMs), skinfold freckling, and cutaneous neurofibromas (cNFs) (13). Intriguingly, despite the significant heterogeneity in the manifestation specifics, the speed of progression, and the severity of complications, the progression of NF1 is typically lifelong, advancing with the individual's age (14). Notably, cognitive impairment, ranging from moderate to severe, afflicts up to 81% of children with NF1. Nearly 40% of these cases fulfill the diagnostic criteria for Attention Deficit Hyperactivity Disorder (13). Beyond the clinical picture, these cognitive impairments impose significant adverse effects on the personal lives of children with NF1, including academic underperformance, behavioral difficulties, and restricted vocational possibilities (15). Therefore, understanding and tackling these complexities is of paramount importance for accurately identifying the unique needs of the patients and providing personalized management in both rehabilitation and educational settings.

Pioneering research highlighted that neurofibromin, partially encoded by the *NF1* gene, can interact with Ras proteins, consequently modulating cellular growth and differentiation (16, 17), via a nf1+/− mouse model, demonstrated that NF1-related learning impairments might be attributed to excessive Ras protein activation. This over-activation enhances the GABA-mediated inhibitory pathway, thereby impeding Long-Term Potentiation (LTP). Sebti underscored the significance of lovastatin as a potent inhibitor of p21Ras/Mitogen-Activated Protein Kinase (MAPK) activity (18). A subsequent study by Li et al. elucidated that lovastatin could attenuate the p21Ras-MAPK activity in nf1 +/− mice, thereby improving LTP and positively influencing the mice's spatial learning and attention (19).

In parallel, cholesterol-reducing drugs, specifically 3-hydroxy-3-methyl-glutaryl (HMG)-CoA reductase inhibitors, have demonstrated promising results in ameliorating cognitive function in various neurological disorders. Notably, statins have showcased potential neuroprotective effects in treating Parkinson's and Alzheimer's patients, such as inhibiting pro-inflammatory molecules and microglial activation, stimulating endothelial nitric oxide synthase, suppressing oxidative stress, reducing the aggregation of α-synuclein, modulating adaptive immunity, and upregulating neurotrophic factors expression (17, 20–23). Intriguingly, statins have also been confirmed to alleviate cognitive impairments in NF1 mice (6, 19). Thereby, statins are considered as potential therapeutics for enhancing cognitive function in children with NF1.

Nevertheless, the current clinical evidence regarding the influence of statins on cognitive dysfunctions in children with NF1 is starkly inconsistent. For example, the research by Bearden postulates that statins can enhance specific memory functions and internalizing behaviors in pediatric patients with NF1 (24). However, this hypothesis was not corroborated in subsequent studies. Only the study by Stivaros suggests that simvastatin can improve physiopathology and social brain region functions based on multiparametric imaging (25). Other studies, such as by Krabt et al. and van der Vaart et al. did not support these findings (26, 27). Consequently, this systematic review and meta-analysis summarized neuropsychological and neurophysiological evidence to illuminate the effects of statin usage on the cognitive function in children with NF 1, based on such clinical outcomes as nonverbal long-term memory, attention concerns, visual spatial memory, daily living functions, and executive functions as evaluative indicators.

## 2. Methods

This investigation strictly aligns with the PRISMA statement, with pre-registration on the PROSPERO platform (#CRD: 42022369072) (28, 29).

### 2.1. Literature sources

Research databases, both Chinese and English, were extensively searched, including China National Knowledge Infrastructure (CNKI), Wangfang Data, PubMed, Embase, Cochrane Library, and Web of Science. The search methodology focused on key terms such as “Neurofibromatosis 1” and “Hydroxymethylglutaryl-CoA Reductase Inhibitors”. A comprehensive search strategy is available in the appendix. The temporal scope was from the establishment of each database until March 31, 2023. References of the selected studies were also traced to supplement relevant literature. The publication language was confined to English.

### 2.2. Criteria for inclusion and exclusion

Criteria for inclusion, formulated based on the PICOS principle (participants, intervention, comparator, outcome, and study design), were as follows:

1. Study population: Individuals definitively diagnosed with NF1. Prior to treatment, no significant statistical variance should exist between the two groups in aspects like gender, age, symptoms, signs, laboratory tests, complications, and comorbidities (*p *> 0.05), indicating comparability.
2. Intervention and comparison: Statins are deployed in the treatment of children with NF-1. The specific dosage and treatment duration are determined by each individual randomized trial. No other lipid-reducing drugs are used, and the medication duration extends for ≥12 weeks.
3. Primary outcome measures: Nonverbal long-term memory, attention issues, visual spatial memory, daily living functions, executive functions.
4. Study type: Published randomized controlled trials (RCTs).

Exclusion criteria: Non-RCTs, studies lacking a description of relevant patient characteristics, duplicate publications, etc.

### 2.3. Screening of literature and data extraction

Two independent researchers undertook literature screening and data extraction. Discrepancies were addressed through discussions involving a third author. EndNote X9 was used for literature management and classification. Initially, the titles and abstracts were screened, with irrelevant ones being discarded. The full texts of potentially relevant studies were then meticulously perused for inclusion determination. If abstracts and titles did not provide adequate information for inclusion or exclusion, the full texts were downloaded and scrutinized according to the set inclusion and exclusion criteria. Extracted content incorporated the article author, publication date, trial sample size, baseline patient data, random sequence generation method, intervention measures in the trial and control groups, specifications, dosage, usage, treatment course, outcome indicators, adverse reactions, etc.

### 2.4. Risk of bias in included studies

The assessment of bias risk within the included studies was meticulously executed by two independent researchers, with any ensuing disagreements settled through discussion involving a third author. We utilized the embedded tool ROB 1.0 in Review Manager (RevMan) 5.4 to assess the quality of the encompassed literature. This evaluation involves five domains: bias stemming from the randomization process, bias induced by deviations from intended interventions, bias originating from missing outcome data, bias in the measurement of the outcome, and bias in the selection of the reported result. The bias risk of each domain was categorized into three levels: “low risk”, “some concerns”, or “high risk”.

### 2.5. Statistical analysis

Statistical analysis was undertaken utilizing RevMan 5.4. The Cochrane *I*^2^ test was employed to gauge the heterogeneity among the included studies. A value of *I*^2^* *< 50% indicated a lack of significant statistical heterogeneity among studies, and thus a fixed-effect model was adopted for the meta-analysis. Conversely, an *I*^2^ value of ≥50% signaled notable statistical heterogeneity among the studies, and therefore the cause of such heterogeneity was explored from a clinical perspective. A random-effect model was invoked for the meta-analysis when necessary. Since the included studies all used scales to assess the outcome indicator for cognitive function improvement in NF1 patients treated with statins, and the scales used in these studies are different, continuous data were represented using a standardized mean difference (MD) with 95% confidence interval (CI). A value of *p *< 0.05 was deemed statistically significant. In cases where more than ten studies were included for the analysis of a specific outcome indicator, publication bias was assessed through a funnel plot.

## 3. Results

### 3.1. Literature search

**Figure 1:**
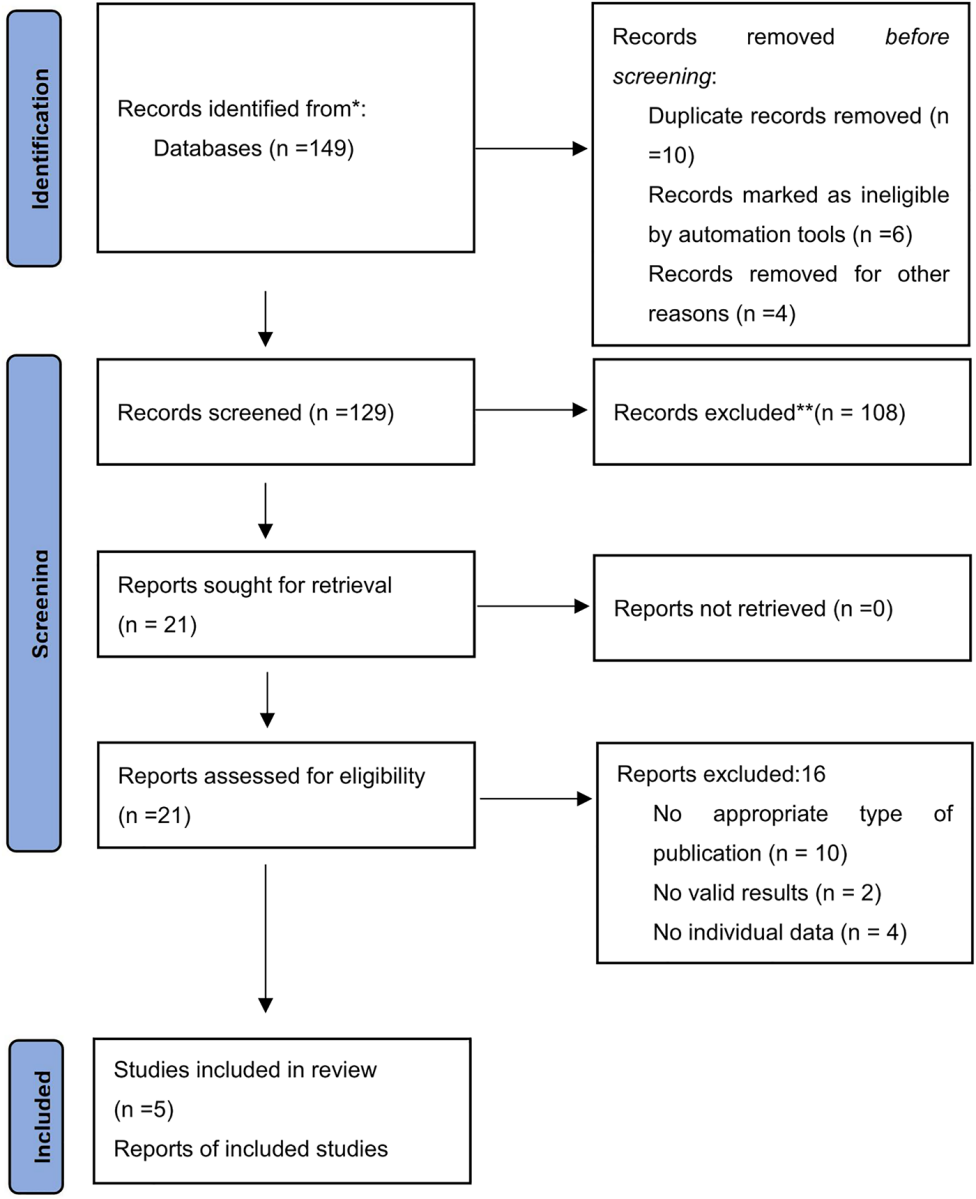
Literature screening process.

A preliminary search yielded a sum of 149 articles. Following an initial evaluation of titles and abstracts, 129 articles were subsequently omitted due to duplicate publications or a failure to meet the exclusion criteria. Upon conducting a comprehensive appraisal of the full texts of the remaining 21 pieces of literature, we further excluded 108 articles due to an absence of recorded data, 16 due to untraceable reports, 10 as a result of incompatible study types, 4 due to deficient data sets, and 2 due to a lack of valid findings. Ultimately, 5 articles were incorporated in the analysis. The entire process of literature acquisition and meticulous screening is illustrated in Figure 1.

### 3.2. Fundamental characteristics of included studies and quality of literature

The included studies encompassed a total of 364 NF1 patients. Of these, three studies involving 87 patients employed Simvastatin, while two studies enrolling 95 patients utilized Lovastatin. All included studies were randomized, double or triple-blind, and employed placebo controls. The overall quality of the 5 included studies was high. Only Van der 2013 and Stivaros 2018 present an unclear risk in a domain, respectively, because Van der 2013 did not provide the registration number in the article, and Stivaros 2018 did not furnish a participant flow diagram. All others were assessed as low risk.

### 3.3. Meta-analysis results

**Figure 2:**
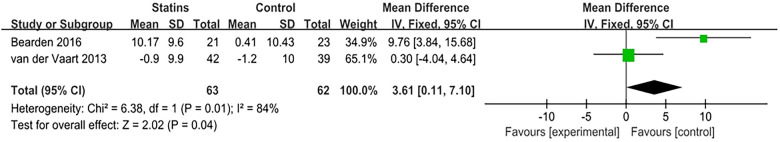
Meta analysis of internalizing problem.

Three studies elucidated the therapeutic effects of statins on internalizing behaviors in NF1 patients, utilizing three evaluation methodologies. In the research conducted by Payne et al. (30), the Behavior Assessment System for Children was harnessed for assessments by both children and parents. The self-assessment results of the children exhibited [MD = −3.2; 95%CI: −9, 2.61; *p* = 0.28], while parental assessment results showed [MD = −1.3; 95%CI: −5.37, 2.77; *p* = 0.53], with both results being negative. However, in the studies by Bearden and van der Vaart et al. (24, 27), the Achenbac Child Behavior Check List (CBCL) scale was employed [95 children; *I*^2^ = 0%, FEM; MD = 3.61, 95%CI: 0.11, 7.10, *p* = 0.04). The results revealed that statins may ameliorate internalizing behavior problems when measured by CBCL. Detailed meta-analysis results are compiled in Figure 2.

**Figure 3:**
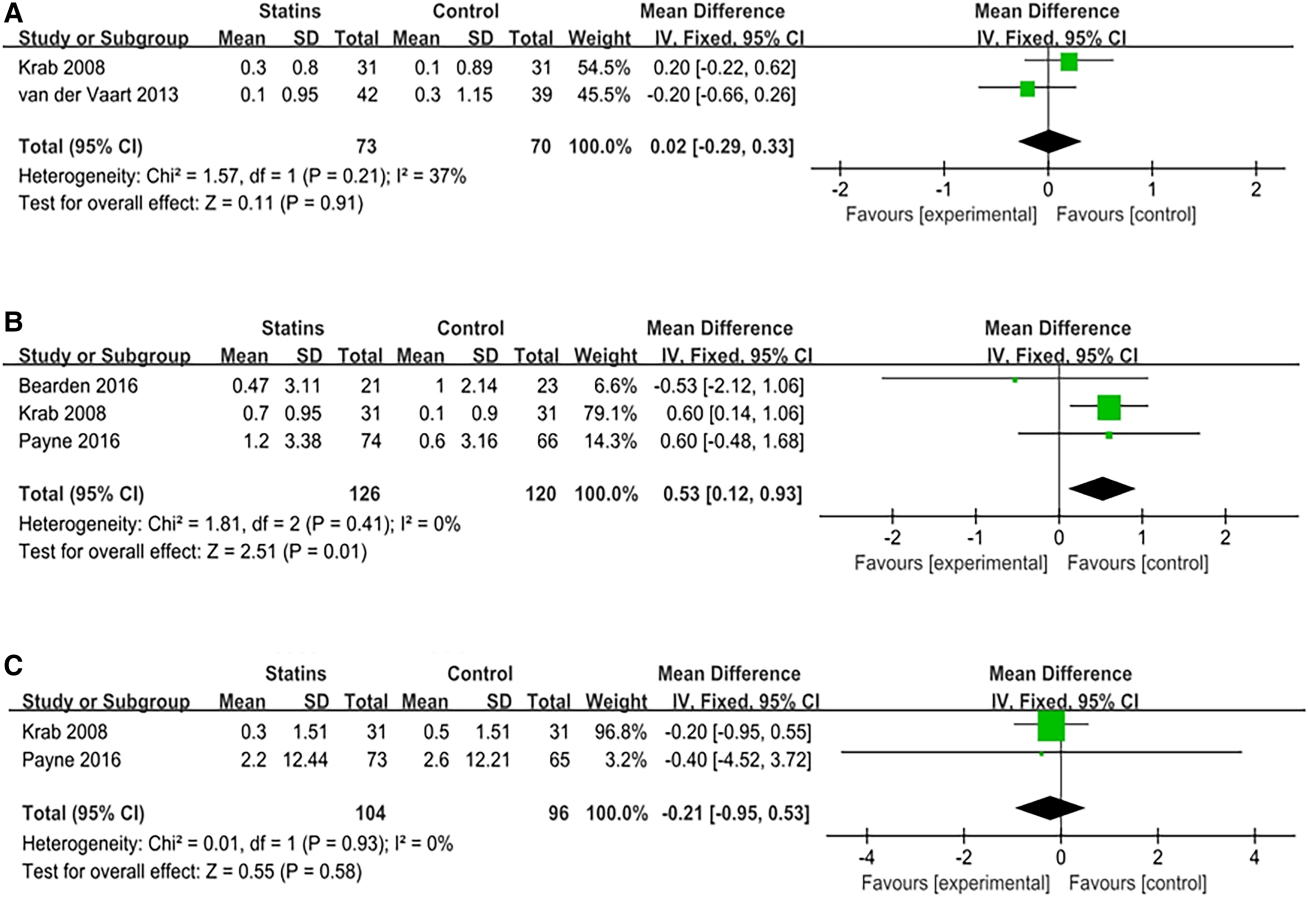
Meta analysis of visual spatial memory. (**A**) Rey-CFT (delayed recall); (**B**) Object assembly; (**C**) Judgment of line orientation test.

Four studies reported on the usage of seven scales to evaluate the potential enhancement effects of statins in visuospatial memory in pediatric patients with NF-1. These scales include the Rey CFT (Rey complex figure test), PAL (Paired Associated Learning) test, BVMT (Brief Visuospatial Memory Test), Block design test, Judgment of line orientation test, Beery VMI test, and Object Assembly test. In the research conducted by Payne et al. (30), the PAL test results showed [MD = 0.20; 95%CI: −5.38, 5.78; *p* = 0.94]. In the study by Krab et al. (26), the Rey CFT (copy) assessment results exhibited [MD = −0.1; 95%CI: −0.70, 0.50; *p* = 0.74]. In the Block design test, the results showed [MD = 0.20; 95%CI: −0.27, 0.67; *p* = 0.40]. In the Beery VMI test showed [MD = 0; 95%CI: −0.4, 0.4; *p* = 1.00]. The BVMT test evaluated both delay and immediate recall, displaying [MD = −1; 95%CI: −8.62, 6.62; *p* = 0.80] for delay recall, and [MD = 0.57; 95%CI: −7.88, 9.02; *p* = 0.90] for immediate recall. Meta-analyses were performed on the remaining three scales, respectively. The meta-analysis of Rey CFT (delayed recall) included 2 studies involving 143 children (*I*^2^ = 0%, FEM) and showed [MD = 0.02; 95%CI: −0.29, 0.33; *p* = 0.91]. Three studies utilized the Object Assembly assessment, comprising a total of 246 children (*I*^2^ = 0%, FEM); the meta-analysis results revealed [MD = 0.53; 95%CI: 0.12, 0.93; *p* = 0.01], which was positive. Two studies utilized the Judgment of line orientation test with an aggregate of 120 children (*I*^2^ = 0%, FEM); the meta-analysis showed [MD = −0.21; 95%CI: −0.95, 0.53, *p* = 0.58]. Meta-analysis results are tabulated in Figure 3. Other results indicated no significant improvement of statins in visuospatial memory in pediatric patients with NF-1, as shown in Table 1.

**Table 1:** Descriptive analysis of mata analysis.

Domain	Study	Measurement	Results [MD(95%CI)]
Attention Problem	Payne 2016	CPT-II (commission errors)	−0.9 [−2.07, 3.87], *p = *0.56
	Payne 2016	CPT-II (omission errors)	−0.7 [−6.24, 4.84], *p = *0.80
	Payne 2016	ADHD (attention)	0.28 [−0.95, 0.01], *p = *0.10
	Payne 2016	Score	0.20 [−0.56, 0.96], *p = *0.60
	Stivaros 2018	Conners questionnaire	5.33[−6.69, 17.53], *p = *0.28
Internalizing Problem	Payne 2016	Behavior assessment system for children, second edition	−3.2 [−9, 2.61], *p = *0.28
	Payne 2016	Behavior Assessment System for Parents, Second Edition	−1.3[−5.37, 2.77], *p = *0.53
Executive Function	Krab 2008	Prism adaption	−0.02 [−0.52, 0.48], *p = *0.93
	Stivaros 2018	Conners questionnaire	0.14 [−0.20, 0.47], *p = *0.42
	Payne 2016	BRIEF GEC	4.04 [−2.51, 10.59], *p = *0.23
	Payne 2016	SOC	−0.50 [−0.96, −0.04], *p = *0.035
	Payne 2016	SST	−27.3 [−57.5,2.9], *p = *0.079
Psychological quality of life	Van der 2013	Full-scale intelligence (WISC-III-NL)	−1.20 [−8.36, 5.96], *p = *0.74
	Vander 2013	CHQ-PF50	0.01 [−0.33, 0.35], *p = *0.95
	Payne 2016	Pediatric quality of life inventory; psychosocial score	−1.90 [−7.67, 3.87], *p = *0.52
Hyperactivity symptoms	Payne 2016	ADHD (hyperactivity symtoms)	2.6 [−2.56, 7.76], *p = *0.32)
	Stivaros 2018	ABC Checklist	3.87 [−9.79, 17.53], *p = *0.58
	Stivaros 2018	Conners questionnaire	0.98 [−12.61, 14.57], *p = *0.89
Visual spatial memory	Krab 2008	Block design	0.20 [−0.27, 0.67], *p = *0.40
	Krab 2008	Beery VMI Test	0 [−0.4, 0.4], *p = *1.00,
	Krab 2008	BVMT delayed	−1 [−8.62.6.62], *p = *0.80
	Krab 2008	BVMT immediate	0.57 [−7.88, 9.02], *p = *0.90)
	Krab 2008	Rey CFT (copy)	−0.1 [−0.70,0.50], *p *= 0.74
	Payne 2016	PAL	0.20 [−5.38, 5.78], *p = *0.94

CPT-II, continuous performance test second edition; CBCL, achenbach child behavior checklist; BVMT, brief vvisuospatial memory test—revised; CHQ-PF50, child health questionnaire–parent form 50; score from test of everyday attention for children; ADHD, attention-deficit/hyperactivity disorder; BRIEF GEC, behavior rating inventory of executive function global executive composite; PAL, paired associated learning; ABC, parent-rated aberrant behaviour checklist.

**Figure 4:**
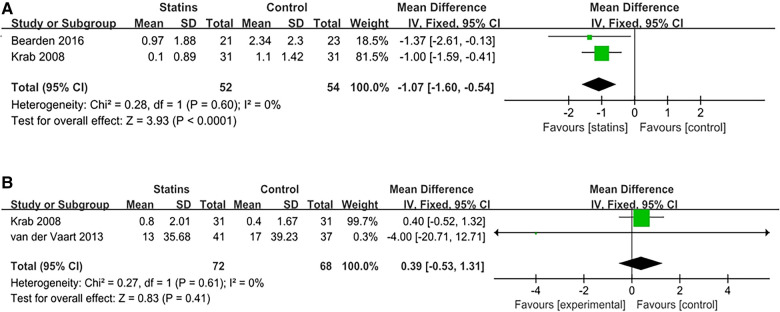
Meta analysis of attention problem. (**A**) Cancellation test; (**B**) Stoop color word test.

The attention capacity in pediatric patients with NF-1 was discussed in all eligible studies, involving a repertoire of six measurement methods: Cancellation Test, Stroop Color Word Test, Test of Everyday Attention for Children score, ADHD (attention) score, Conners (attention) score, and CPT-II (Continuous Performance Task-II) scale. The research led by Payne et al., (30), utilized two evaluative measures: Test of Everyday Attention for Children score (MD[95%CI = 0.2[−0.56,0.96], *p* = 0.60), ADHD (attention) score (MD[95%CI = 0.28[−0.95,0.01], *p* = 0.10). The Conners score was adopted in the study by Stivaros et al., (25) [MD = 5.33; 95%CI: −6.69, 17.53, *p* = 0.28], to evaluate enhancements in patients' attention. In the context of the CPT-II scale employed in the study by Payne et al. (30), both Commission errors [MD = −0.9; 95%CI: −2.07, 3.87; *p* = 0.56] and Omission errors [MD = −0.7; 95%CI: −6.24, 4.84; *p* = 0.80] were assessed, yielding negative outcomes. The remaining 2 evaluative approaches were reported in a multitude of articles, and thus meta-analyses were conducted. The synthesized results are as follows: Cancellation Test [2 studies, 52 children, *I*^2^ = 0%, FEM; MD = −1.07, 95%CI: −1.60, −0.54, *p* < 0.01), and Stroop Color Word Test (2 studies, 72 children, *I*^2^ = 0%, FEM; MD = 0.39, 95%CI: −0.53, 1.31, *p* = 0.41). Given the low heterogeneity of these tests, a fixed-effects model was employed. Statin administration demonstrated improvements only in the Cancellation Test when compared with the placebo. Further meta-analysis results are available in Table 1. Other research outcomes and meta-analysis findings indicate that statins did not significantly ameliorate attention capacity, as elucidated in Figure 4.

In evaluating the progression in hyperactivity symptoms in pediatric patients with NF-1 post statin administration, two studies were included, involving three assessment scales: ADHD scale, Conners scale, and Parent-rated Aberrant Behaviour Checklist (ABC) scale. Payne et al. used the ADHD scale [MD = 2.6; 95%CI: −2.56,7.76; *p* = 0.32] (30). Stivaros et al. used Conners scale [MD = 0.98; 95%CI: −12.61,14.57; *p* = 0.89] and ABC scale [MD = 3.87, 95%CI: −9.79,17.53, *p* = 0.58] (25). The results suggest that statins did not improve hyperactivity symptoms in pediatric patients (Table 1).

The executive function was mentioned in three studies, involving three evaluation methods: Prism adaption score, BRIEF GEC (Behavior Rating Inventory of Executive Function Global Executive Control) score, Conners executive function score, SOC (Stockings of Cambridge) score, and SST (Stop Signal Task) score. As each evaluation method was not repeatedly reported, no meta-analysis was conducted. In the study of Krab et al. (26), Prism adaption was used for evaluation [MD = −0.02; 95%CI: −0.52, 0.48, *p* = 0.93]. In the study of Stivaros et al. (25), the Conners (Executive function) scale was used for evaluation [MD = 0.14; 95%CI: −0.20, 0.47, *p* = 0.42]. In the study of Payne et al. (30), 3 scales were used to assess children's executive function [BRIEF GEC: MD = 4.04, 95%CI: −2.51, 10.59, *p* = 0.23; SOC: MD = −0.50, 95%CI: −0.96, −0.04, *p* = 0.035; SST: MD = −27.3, 95%CI: −57.5, 2.9, *p* = 0.079]. We have summarized the results of each study, and all the results indicate that statin administration did not significantly improve children's executive function, as shown in Table 1.

Whether statin medication administration could improve the quality of life of pediatric patients with NF-1 was reported in three studies, involving four evaluation scales: the CBCL scale, YASR (Achenbach Young Adult Self-Report), the CHQ-PF50 Scale (Child Health Questionnaire–Parent Form 50), and the Pediatric Quality of Life Inventory-Psychosocial Score scale. In the study conducted by van der Vaart et al., (27), the CHQ-PF50 scale analysis showed [MD = 0.01; 95%CI: −0.33,0.35, *p* = 0.95]; the Full-Scale Intelligence (WISC-III-NL) analysis result was [MD = −1.20; 95%CI: −8.36, 5.96; *p* = 0.74], while the Teacher-Rated School Performance (CBCL) analysis result showed [MD = 0.1; 95%CI: −0.95,1.15; *p* = 0.85]. In the study by Payne et al., (30), the Pediatric Quality of Life Inventory-Psychosocial Score Scale was applied, separately evaluated by both children and parents. The self-assessment result of the children was [MD = −1.90; 95%CI: −7.67, 3.87; *p* = 0.52], whereas the parental assessment result came out to be [MD = 2.90; 95%CI: −2.74, 8.54; *p* = 0.31], with both findings being negative. Lastly, in the research conducted by Bearden et al., (24), the Thought Problems (CBCL) evaluation result emerged as [MD = −2.16; 95%CI: 7.03, −2.71; *p* = 0.38], and the Social Problem (CBCL) evaluation result presented as [MD = 0.55; 95%CI: −4.17, 5.27; *p* = 0.82]. Cumulatively, other results and the corresponding meta-analysis findings showed that the administration of statin medications did not significantly improve life quality, as detailed in Table 1.

## 4. Discussion

In synthesizing available clinical evidence through a systematic review and meta-analysis, it becomes apparent that, following a period of 12–14 weeks, statin administration yielded discernible improvements in internalizing problem, visual memory (measured by object assembly) and attention difficulties (measured by Cancellation Test) in pediatric patients with NF-1, in comparison to a placebo group. However, no significant improvements in other cognitive domains were observed.

As for the underlying mechanisms responsible for these effects, statins are posited to hold potential cognitive advantages for NF-1 patients, but the exact therapeutic effects remain unknown. Past studies such as that by Acosta (31) reported the initial results of lovastatin treatment in NF-1 patients, suggesting potential benefits for verbal and non-verbal memory, but no statistically significant effects were noted in terms of attention and alertness.

The current body of research suggests that the cognitive enhancements witnessed in NF-1 patients following statin treatment might be attributed to three main factors. Primarily, lovastatin may exhibit a positive effect on certain known structural domains that are typically compromised in NF-1 patients. Studies by Greicius et al. and Chabernaud et al. (32, 33) revealed that lovastatin, under medicated conditions, enhanced the long-term positive RSFC (resting state functional connectivity) within the core regions of the Default Network (DN), specifically the anterior medial prefrontal cortex and the posterior cingulate cortex (PCC). This leads to the conjecture that statins may augment cognitive functioning in NF-1 patients through ameliorating DN functional connectivity.

Secondly, a randomized trial by Li et al. (19) has verified that lovastatin inhibits the hyperactive RAS pathway, thereby bolstering synaptic plasticity and rectifying learning and attention deficits in NF1 mouse models. The conclusions drawn by Cui et al. (34) and Costa et al. (17) indicate that learning difficulties associated with NF1 may stem from excessive Ras activation, resulting in an elevated release of GABA in the hippocampus. The inhibitory nature of GABA on hippocampal synaptic plasticity could result in deficits in hippocampal-dependent learning.

Lastly, cognitive deficits attributed to certain other neurological diseases may potentially be mitigated by statins. It is widely accepted that many neurological diseases induce oxidative stress. A growing body of research (35, 36) posits that Ras directly regulates the production of ROS (reactive oxygen species), implying a potential link between NF-1 and oxidative stress induction. Given that oxidative stress responses can impair cognitive function (37–39), it is plausible to hypothesize that NF-1-induced oxidative stress could serve as another contributor to cognitive decline in affected patients. Numerous studies (20, 21) indicated that statins may provide neuroprotection by impeding pro-inflammatory molecules and microglial activation, stimulating endothelial nitric oxide synthase, and mitigating oxidative stress.

Translating the given evidence into effective treatments is challenging. Our meta-analysis shows that statin therapy does not significantly improve cognitive functions in children with NF-1. This can potentially be attributed to a multitude of factors. Firstly, cognitive deficits and behavioral irregularities in NF-1 patients may stem from diverse pathways. The proportional contribution of each mechanism may vary among individuals, thereby introducing inconsistencies in assessment and evaluation. Secondly, in terms of attention-related issues in NF-1 children, there were significant disparities between test outcomes and questionnaire evaluations. This apparent instability in scoring could echo the inherent variability within the NF-1 population, which may be ignored by evaluators (e.g., educators, parents). Lastly, when extrapolating trials from mouse models to NF-1 children, we must acknowledge the behavioral ecological disparities between mice and children. A paucity of explicit evidence of parallels between human neuroanatomical structures and those in NF-1 mouse models must also be recognized (40, 41).

In a clinical investigation employing lovastatin on NF-1 pediatric patients conducted by Ullrich et al. (42), a portable, computerized task labeled the “Arena Maze” was deployed to assess potential enhancements in the visuospatial memory in children. The results suggest that lovastatin treatment failed to amplify the children's spatial learning capabilities. This might be attributable to the children's pre-existing familiarity with digital products and the modest challenge the test posed. Furthermore, research by Torres Nupan et al., (43) infers that the benefits of statins in humans remain to be conclusively verified. Statin administration might even inflict detrimental effects on cognitive functions in patients, such as exacerbating cognitive impairment and accelerating memory decline (44).

Even though hyperactivation of Ras protein and elevated GABA release are typically viewed as the principal mechanisms instigating cognitive deterioration in NF-1 children, biomarkers linking neurofibromin expression to cognitive outcomes are yet to be established (45). This casts a shadow of doubt on the presumed action mechanism of statins. Lovastatin did not significantly influence functions controlled by the prefrontal-striatal circuit, such as spatial working memory. Given that this trial undertook multiple uncorrected statistical comparisons, prudence should be exercised in interpreting such preclinical findings. An intriguing observation during the trial was the decrease in blood cholesterol levels in NF-1 children receiving lovastatin, thereby indicating the inhibitory effect of statin on the HMG-CoA reductase pathway in the liver. Nevertheless, the existence of the blood-brain barrier presents a challenge. Even though statins, due to their lipophilic nature, can traverse this barrier freely, the maximum dosage prescribed for pediatric patients with NF-1 might still fall short of exerting a therapeutic effect on cerebral functionality.

This research is subject to certain limitations. A notable one is the restricted number of studies included and a paucity of case numbers, which have consequently resulted in a more constrained evidence base for this investigation. This situation rendered us incapable of testing for publication bias, which potentially dilutes the strength of our conclusions. Moreover, the assessment scales employed in the studies included were markedly inconsistent, affecting the trustworthiness of our combined outcomes. We advocate for future clinical RCTs to adopt more standardized assessment scales to aid clinical decision-making by practitioners. Future well-designed, large-sample, clinical RCTs are desired to amplify our understanding of the influence of statin drugs on cognitive functions in children with NF-1.

## 5. Conclusion

The small sample sizes and the difference in scales used in the included studies compromise the reliability of our results. Despite these limitations, the existing evidence still unveils that statins are ineffective in improving cognitive impairments in pediatric patients with NF-1.

## Data Availability

The original contributions presented in the study are included in the article/Supplementary Material, further inquiries can be directed to the corresponding author.
